# Microbial community dynamics in replicate anaerobic digesters exposed sequentially to increasing organic loading rate, acidosis, and process recovery

**DOI:** 10.1186/s13068-015-0309-9

**Published:** 2015-08-19

**Authors:** Xavier Goux, Magdalena Calusinska, Sébastien Lemaigre, Martyna Marynowska, Michael Klocke, Thomas Udelhoven, Emile Benizri, Philippe Delfosse

**Affiliations:** Environmental Research and Innovation (ERIN) Department, Luxembourg Institute of Science and Technology (LIST), 41 rue du Brill, 4422 Belvaux, Luxembourg; Laboratoire Sols et Environnement, UMR 1120, Université de Lorraine, 2 avenue de la Forêt de Haye, TSA 40602, 54518 Vandœuvre-lès-Nancy, France; Laboratoire Sols et Environnement, UMR 1120, INRA, 2 avenue de la Forêt de Haye, TSA 40602, 54518 Vandœuvre-lès-Nancy, France; Department Bioengineering, Leibniz Institute for Agricultural Engineering Potsdam-Bornim, Max-Eyth-Allee 100, 14469 Potsdam, Germany; Fachbereich VI- Raum- und Umweltwissenschaften, Umweltfernerkundung & Geoinformatik, Universität Trier, 54286 Trier, Germany

**Keywords:** Anaerobic digestion, Volatile fatty acids intoxication, Process recovery, 16S rRNA gene-based T-RFLP, High-throughput 16S rRNA amplicon sequencing, Microbial ecology

## Abstract

**Background:**

Volatile fatty acid intoxication (acidosis), a common process failure recorded in anaerobic reactors, leads to drastic losses in methane production. Unfortunately, little is known about the microbial mechanisms underlining acidosis and the potential to recover the process. In this study, triplicate mesophilic anaerobic reactors of 100 L were exposed to acidosis resulting from an excessive feeding with sugar beet pulp and were compared to a steady-state reactor.

**Results:**

Stable operational conditions at the beginning of the experiment initially led to similar microbial populations in the four reactors, as revealed by 16S rRNA gene T-RFLP and high-throughput amplicon sequencing. *Bacteroidetes* and *Firmicutes* were the two dominant phyla, and although they were represented by a high number of operational taxonomic units, only a few were dominant. Once the environment became deterministic (selective pressure from an increased substrate feeding), microbial populations started to diverge between the overfed reactors. Interestingly, most of bacteria and archaea showed redundant functional adaptation to the changing environmental conditions. However, the dominant *Bacteroidales* were resistant to high volatile fatty acids content and low pH. The severe acidosis did not eradicate archaea and a clear shift in archaeal populations from acetotrophic to hydrogenotrophic methanogenesis occurred in the overfed reactors. After 11 days of severe acidosis (pH 5.2 ± 0.4), the process was quickly recovered (restoration of the biogas production with methane content above 50 %) in the overfed reactors, by adjusting the pH to around 7 using NaOH and NaHCO_3_.

**Conclusions:**

In this study we show that once the replicate reactors are confronted with sub-optimal conditions, their microbial populations start to evolve differentially. Furthermore the alterations of commonly used microbial parameters to monitor the process, such as richness, evenness and diversity indices were unsuccessful to predict the process failure. At the same time, we tentatively propose the replacement of the dominant *Methanosaeta* sp. in this case by *Methanoculleus* sp., to be a potential warning indicator of acidosis.

**Electronic supplementary material:**

The online version of this article (doi:10.1186/s13068-015-0309-9) contains supplementary material, which is available to authorized users.

## Background

Anaerobic digestion (AD and syn. biomethanation) of biomass, including wastewater, agro-food residues, municipal solid waste, and energy crops, is not only regarded as a promising source of renewable energy, but also generates environmental benefits, e.g. reduction of greenhouse gas emissions, manure odour and pathogens [1, 2] and contributes to the recovery of essential nutrients (N, P, and K). The main products of the AD process are the biogas and the digestion residue. Biogas is composed of methane (CH_4_), carbon dioxide (CO_2_) and trace gases such as H_2_S, NH_3_ and H_2_, and it can be combusted in combined heat and power units (CHP) to provide electricity and heat. Alternatively, by eliminating CO_2_ and trace gases, methane can be upgraded to reach the natural gas quality and be injected into the gas grid or used as transport fuel. Digestion residue being rich in nutrients is gaining reputation as a fertilizer in agriculture [3].

The AD process is conducted by different microbial groups interacting to decompose the organic matter into minerals and simple molecules. During the first stage called hydrolysis, facultative or obligatory anaerobic fermenting microorganisms decompose proteins, fats and polysaccharides into soluble compounds (i.e. amino acids, long-chain fatty acids and sugars). In continuation, during the acidogenic stage, acidogenic bacteria convert these by-products into volatile fatty acids (VFAs), CO_2_, H_2_, and alcohols. Furthermore, VFAs, CO_2_ and H_2_ are transformed by acetogenic microorganisms to produce acetate (acetogenesis stage). Finally during the methanogenesis stage, acetate and H_2_, CO_2_ are used, respectively, by the acetotrophic (syn. acetoclastic) and hydrogenotrophic methanogenic archaea to produce methane [4]. Even though the major paths of the AD process are well described, the complexity of the microbial activities, competition, and syntrophism are not well understood [5, 6]. Importantly, the performance of an AD reactor is closely linked to the structure and dynamics of its microbial community (microbiome) [7]. Consequently, the importance of understanding the AD microbiome and the need of establishing microbial indicators of process performance are currently considered as key research subjects towards the improvement of the biomethanation process and the understanding of the process imbalance [6].

Acidification of anaerobic reactors (acidosis) results from an accumulation of VFAs due to an overload of the feeding substrate or the use of a rapidly degradable substrate and sometimes due to the temperature drop [8, 9]. VFAs accumulation directly reflects the kinetic imbalance between their production by fermentative and acidogenic bacteria and their consumption by a combined effort of acetogenic bacteria and methanogenic archaea [10]. Acidosis is the most common process failure taking place in many AD reactors [9, 11] and for which the biogas unit owners seek advice and recommendation on how to quickly and efficiently recover the process (personal interactions with the biogas plants owners in the Greater Region including Luxembourg, and partially Belgium, France and Germany). In general, acidosis is not easy to prevent [12], and high VFAs concentration in the reactors do not necessarily result in pH decrease [13]. However, due to their high sensibility to the increased VFAs concentrations and pH changes, archaeal communities in AD reactors facing acidification are quickly inhibited, leading to a decreased methane production [14]. As a consequence, acidosis represents an important loss for the biogas plants both in terms of reduced methane production and an acidic digestion residue, which do not meet the requirements for optimal fertilization anymore [15, 16]. For these reasons, it is important on the one hand to efficiently predict and prevent acidosis and on the other hand, to quickly restart the process once the acidosis takes place [17]. Indeed, a poor biogas quality (low CH_4_ content) usually results in stopping the CHP unit with heat no longer being provided to the AD reactor, thus further worsening the process status of the plant. While recent studies have brought more evidence about the changes in microbial communities during the process perturbation related to VFAs accumulation [9, 13, 18, 19], little is known about the individual sensitivity of the reactors and the capability of the process to recover when the pH is brought back to neutrality.

**Fig. 1:**
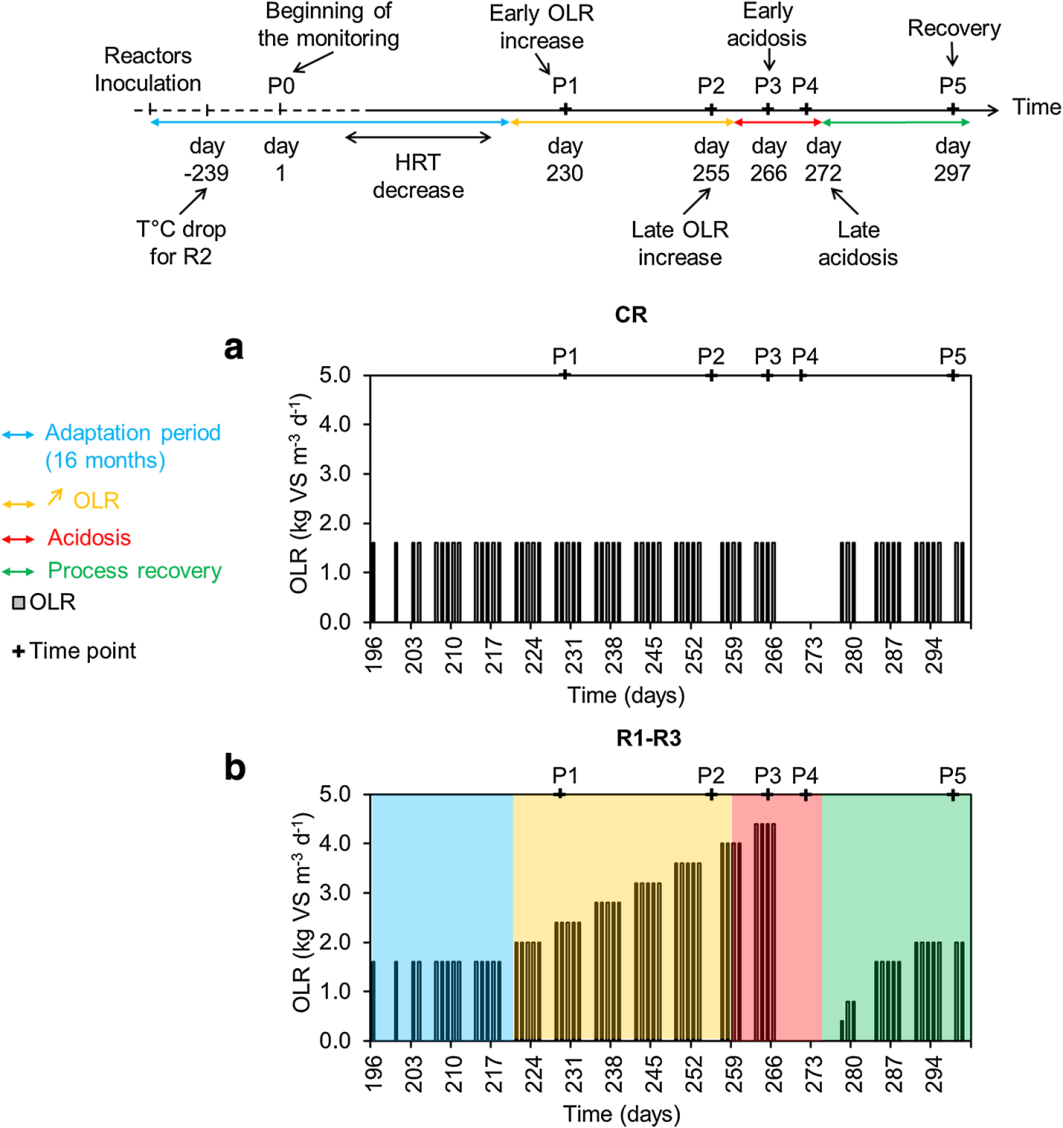
Schematic diagram of the experimental feeding campaign. Progress of the organic loading rate (OLR) over time for the cautiously fed reactor (CR, **a**) and the test reactors (R1–R3, **b**); OLR: organic loading rate (*bars*) in kg VS m^−3^ d^−1^; *P0* (beginning of the monitoring), *P1* (early OLR increase), *P2* (late OLR increase), *P3* (early acidosis), *P4* (late acidosis), and *P5* (recovery) are the sampling periods chosen for microbial community analyses

Therefore, this study had two objectives. First, to investigate the assumption that an increasing content of VFAs in some reactors leading to pH drop, directly influences the microbial communities (what is reflected by a decreased methane production), we gradually increased the organic loading rate (OLR) in three test reactors (R1, R2 and R3), while a control reactor (CR) was constantly cautiously fed (Fig. 1). Second, to establish potential (microbial) warning indicators of a process failure, we characterized the dynamics of the microbial (bacterial and archaeal) communities for six sampling periods P0–P5 in relation to physicochemical parameters in the continuously (completely) stirred tank reactors (CSTRs) sequentially exposed to (1) an increasing OLR of sugar beet pulp, (2) acidosis, and (3) process recovery. Microbial communities in the CSTRs were studied by means of molecular techniques including, 16S rRNA gene-based terminal restriction fragment length polymorphism (T-RFLP) and 16S rRNA high-throughput amplicon sequencing (HTS). In addition, common process parameters such as pH, total solids (TS), volatile solids (VS), alkalinity, ammonium–nitrogen (NH_4_–N) and biogas production and quality were monitored and correlated with the dominant microbes and the state of the AD process.

## Results and discussion

### Operational parameters and anaerobic process performance

**Fig. 2:**
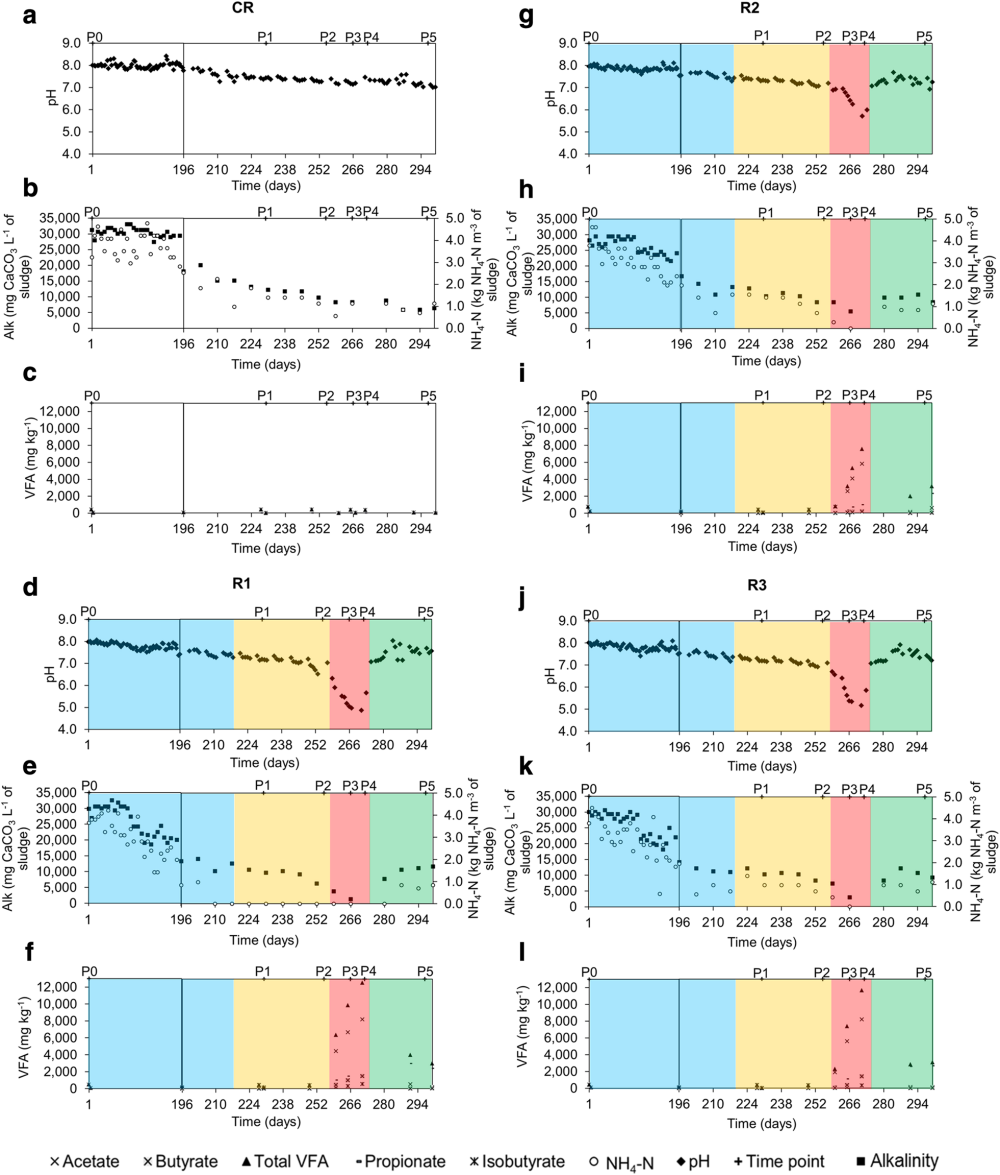
Dynamics of pH, alkalinity, ammonium–nitrogen and volatile fatty acids contents in the sludge for each reactor over time. Progress over time for CR, R1, R2, and R3, respectively, of pH—**a**, **d**, **g**, **j**; total alkalinity (Alk., mg CaCO_3_ L^−1^ of sludge) and ammonium–nitrogen content (NH_4_–H, kg NH_4_–N m^−3^ of sludge)—**b**, **e**, **h**, **k**; volatile fatty acid content (VFA, mg kg^−1^)—**c**, **f**, **i**, **l**; CR is the cautiously fed reactor; R1, R2 and R3 are the reactors exposed to increasing organic loading rate; P0–P5 are the sampling periods chosen for microbial community analyses

The reactors were previously acclimated for 16 months to adapt to a mono-substrate, sugar beet pulp, and to reach a steady-state with a final hydraulic retention time (HRT) of 28.5 days (Fig. 1). CR was constantly and cautiously fed with an OLR of 1.60 kg m^−3^ day^−1^ of sugar beet pulp, assuring a steady-state process with sludge parameters in the range of pH 7.1–7.6, alkalinity of 8824.9 ± 2.5 mg CaCO_3_ L^−1^ of sludge, NH_4_–N under 1.5 kg NH_4_–N m^−3^ (Fig. 2a–c) and the TS and VS contents fluctuating between 1.9–2.4 % and 60–67 %, respectively. For the three test reactors R1–R3 and from the beginning of the OLR increase (sampling period P1), a substantially low sludge content in TS, between 2.5 and 4.1 %, resulted in their high sensitivity to VFA accumulation (on average 10,589 ± 2678 mg L^−1^) and pH decrease (on average 5.2 ± 0.4) (Fig. 2). This took place at an OLR of 4.5 kg m^−3^ day^−1^. Alkalinity also decreased from around 10,132 mg CaCO_3_ L^−1^ of sludge at P1 to approximately 1471; 5393 and 2942 for R1, R2 and R3, respectively, during the late OLR increase (P2). At P1, acetate was the dominant VFA in overfed reactors and its concentration in the sludge was 409 ± 26 mg kg^−1^. During the late acidosis (P4), the concentration in total VFAs reached approximately 12,000 mg kg^−1^ for R1 and R3, and 8000 mg kg^−1^ for R2. At this stage, acetate, propionate, and isobutyrate were the dominant VFAs and accounted for 70 % ± 6, 11 % ± 1 and 3 % ± 1 of total VFAs, respectively. Even though a propionate/acetate ratio above 1.4 is considered as one of the most appropriate indicators of process imbalance [20], for the three overfed reactors it did not exceed 0.17 ± 0.0, what could prevent the right diagnosis of the reactor state if only relying on VFAs measurement. Indeed, different authors previously reported that different reactors have their specific VFAs levels to serve as process indicator, and the conditions that are considered stable for one reactor may not be optimal for another [13, 21, 22].

**Fig. 3:**
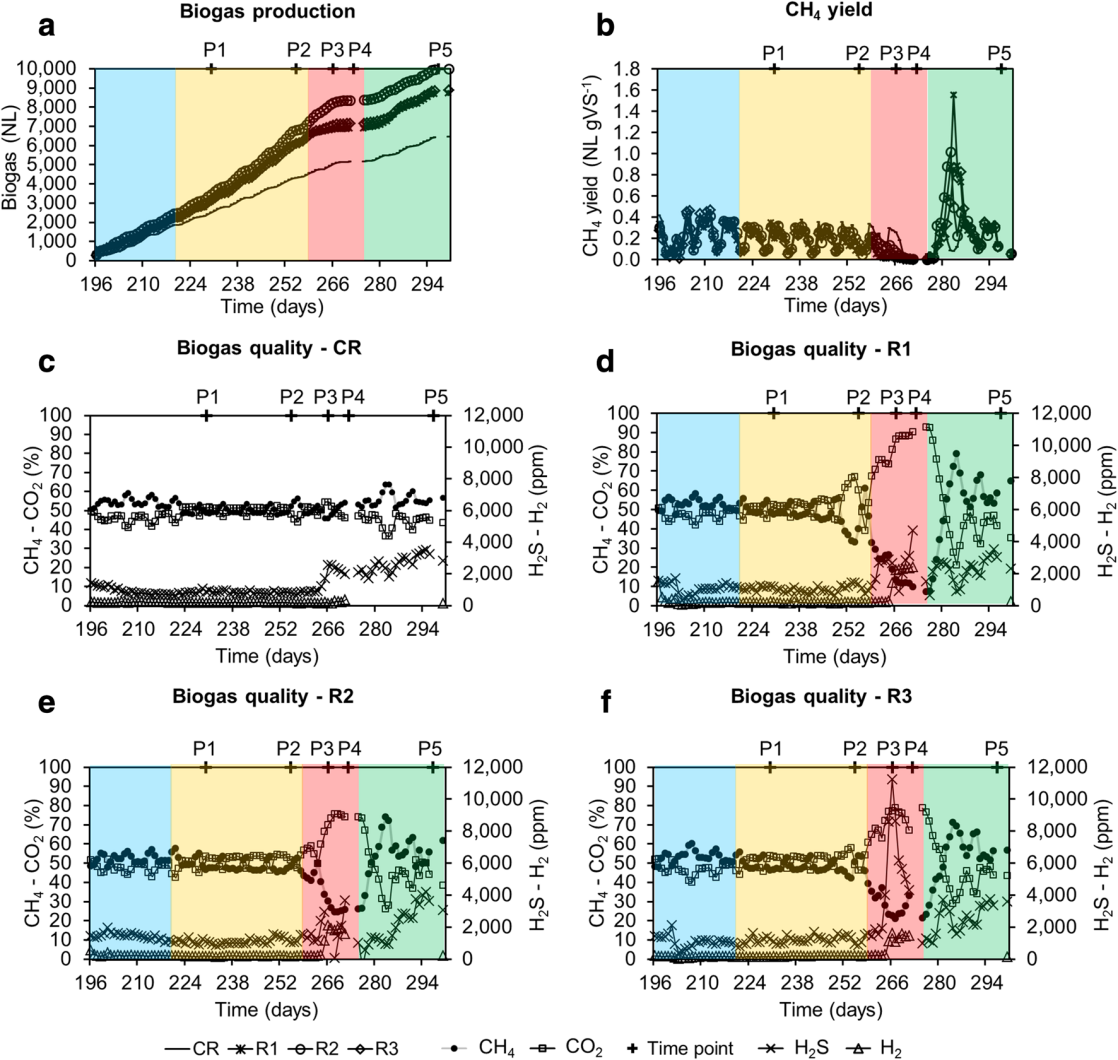
Progress of the biogas production and composition over time for the four reactors. Biogas production for the four reactors (in NL) (**a**); CH_4_ yield for the four reactors (in NL g VS^−1^) (**b**); biogas quality (CH_4_ and CO_2_ in %, H_2_S and H_2_ in ppm) for CR, R1; R2 and R3 (**c**, **d**, **e** and **f**, respectively); CR is the cautiously fed reactor; R1, R2 and R3 are the reactors exposed to increasing organic loading rate; P1–P5 are the sampling periods chosen for microbial community analyses; gaps in the *curves* indicate missing data due to measurement failure

Significant differences in biogas production and composition were also observed during the late acidosis phase (Fig. 3), with methane constituting roughly 9, 25, and 32 % of the biogas for R1, R2 and R3, respectively, while it equalled a minimum of 55 % for CR. Moreover, peeks of increased H_2_ production (>2000 ppm) were detected for R1–R3 indicating a decoupling between the H_2_-producing and H_2_-consuming bacteria (Fig. 3d–f).

Following the acidosis, a process recovery (restoration of biogas production with average methane content above 50 %; P5) was promoted by an artificial pH increase, what led to a decrease of total VFAs to a level of 4000 mg kg^−1^ for R1, 2000 mg kg^−1^ for R2 and 2800 mg kg^−1^ for R3 at the sampling period P5 (Fig. 2f, i, l). A significant increase in methane content in the biogas was correlated with acetate consumption, what could indicate the re-activation of acetoclastic methanogenesis. However, at the same time propionate was the most dominant VFA (75 and 90 % of total VFAs for R1 and R2–R3, respectively) and the propionate/acetate ratio was above 12.5 ± 6.4 during the recovery stage. Incapacity to quickly re-consume propionate might point to the fact that acetogenic bacteria were the one most affected by the experimental conditions examined in this study. Moreover, syntrophic propionate degradation is a thermodynamically very unfavourable reaction (ΔG^0′^ equals +72 kJ mol^−1^), that occurs only when an optimized balance (pH_2_ below 10^−5^ atm) between H_2_-producing (acetogens) and H_2_-consuming (mostly hydrogenotrophic methanogens and homoacetogens) microbes exists [23].

### Comparison of the 16S rRNA gene-based T-RFLP and high-throughput 16S rRNA amplicon sequencing results

**Fig. 4:**
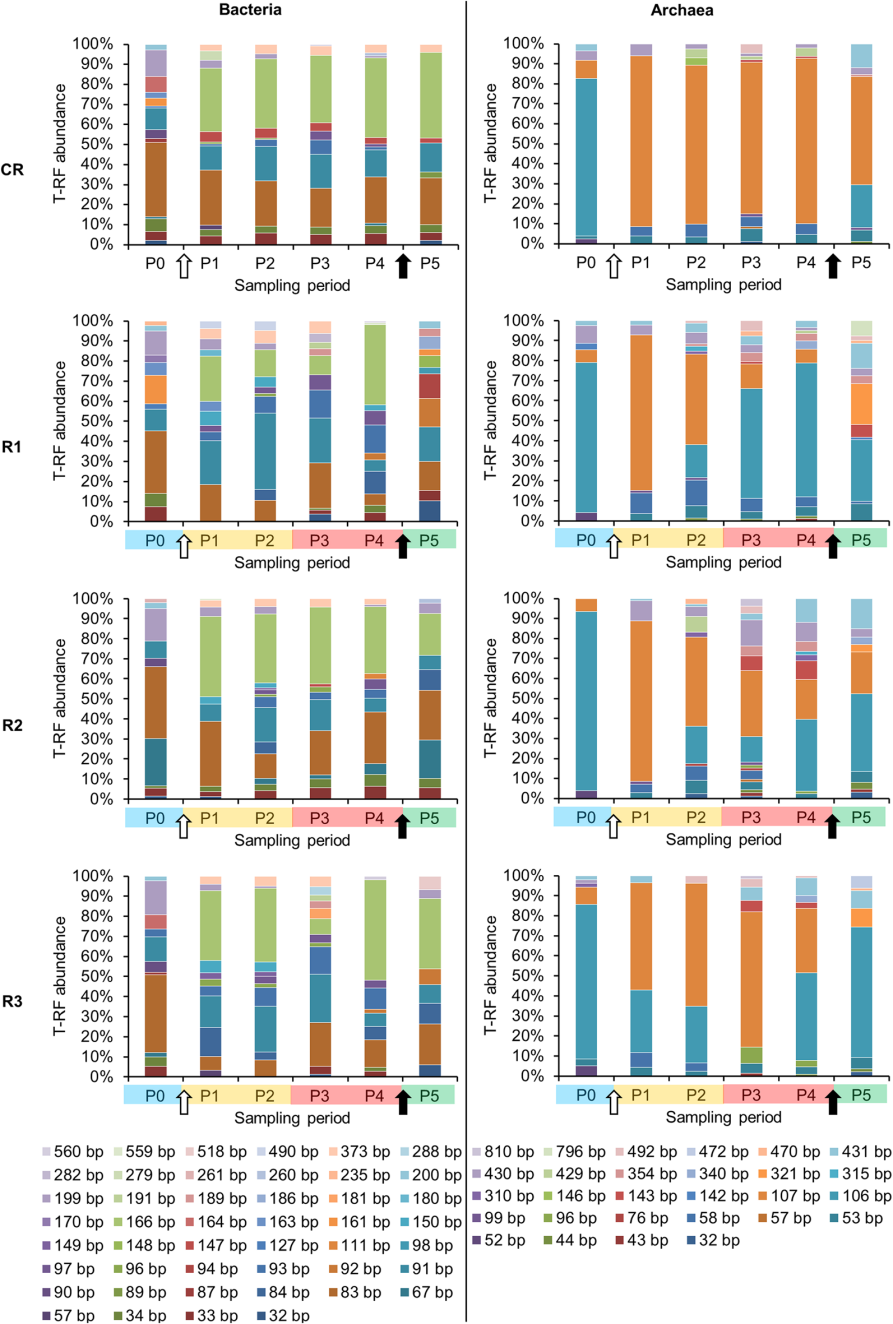
Bacterial and archaeal community structure dynamic over time as assessed by 16S rRNA gene-based T-RFLP analysis. P0–P5 are the six sampling periods chosen for microbial community monitoring; CR is the cautiously fed reactor; R1, R2 and R3 are the reactors exposed to increasing organic loading rate; T-RFs are characterized by their length in base pairs (bp); the *white arrow* represents the initiation of the hydraulic retention time decrease; the *black arrow* represents the onset of the starving period

**Fig. 5:**
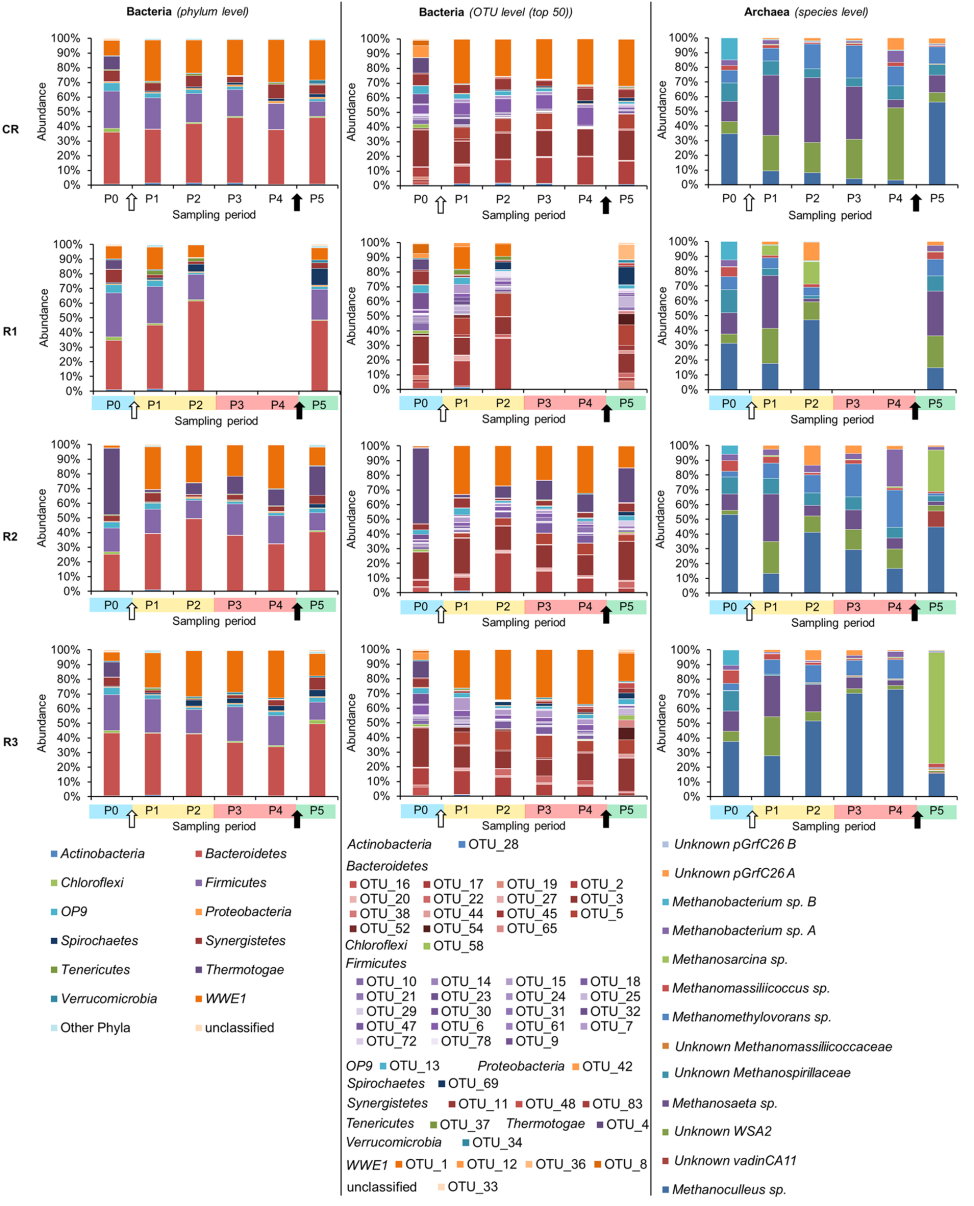
Bacterial and archaeal diversity dynamic over time as assessed by high-throughput 16S rRNA amplicon sequencing. P0–P5 are the sampling periods chosen for microbial community monitoring; CR is the cautiously fed reactor; R1, R2 and R3 are the reactors exposed to increasing organic loading; results are presented at the phylum level and the OTU level (top 50) for bacteria and at the species level for archaea; gaps for R1 at sampling periods P3 and P4 were due to the poor quality of extracted DNA during severe acidosis; the *white arrow* represents the initiation of the hydraulic retention time decrease; the *black arrow* represents the onset of the starving period

**Fig. 6:**
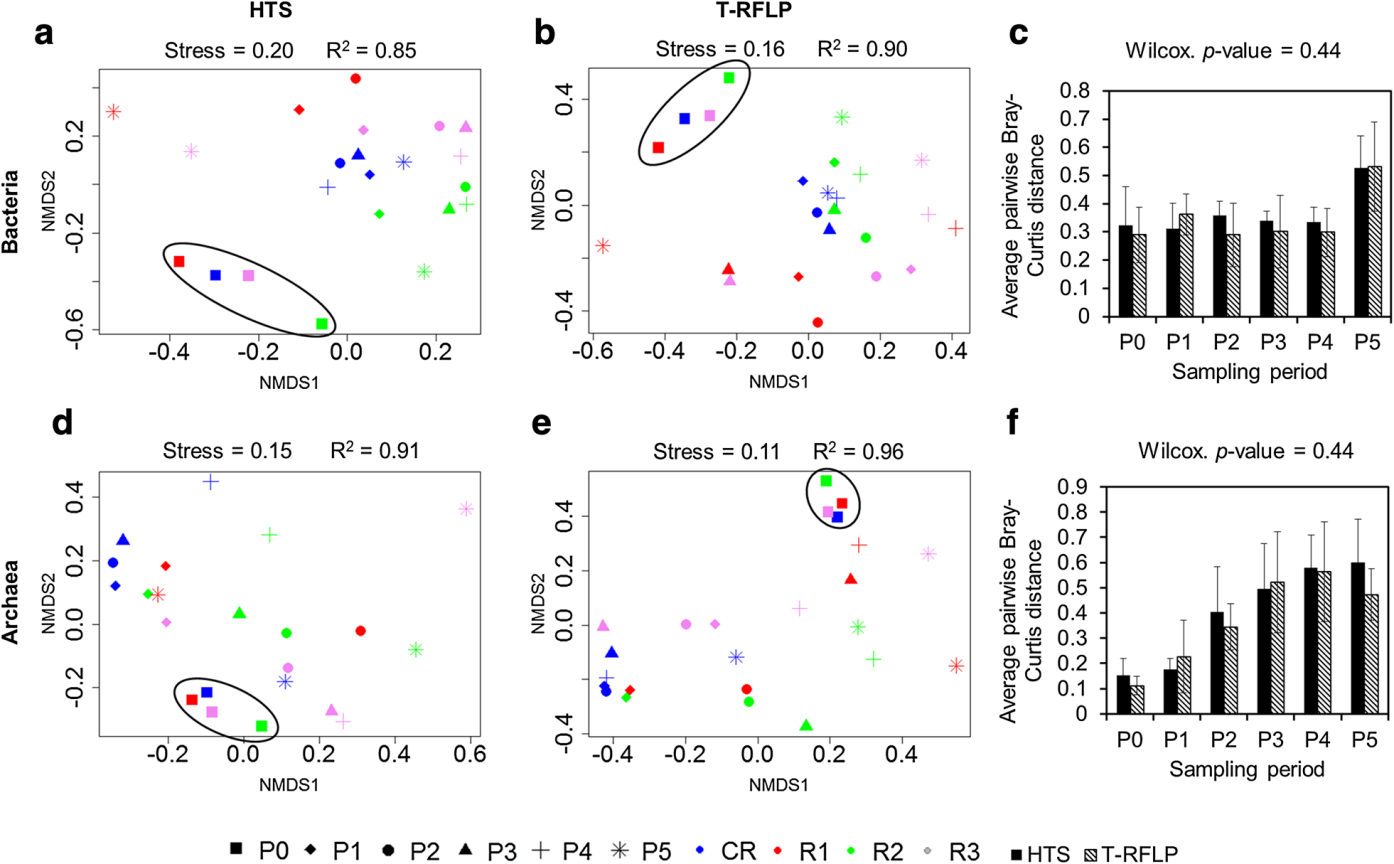
Non-metric multidimensional scaling ordination diagrams of temporal variations in bacterial and archaeal community structures. The ordination is based on Bray–Curtis similarity matrices of the relative abundance data obtained from high-throughput amplicon sequencing (**a** and **d**) and T-RFLP (**b** and **e**) for the bacterial and archaeal community, respectively; **c** and **f** show the average pairwise Bray–Curtis distance for the four reactors at the different sampling periods based on the high-throughput amplicon sequencing and T-RFLP results for, respectively, the bacterial and archaeal community; the *black ellipses group* sampling period P0 (steady-state process) for each reactor

Microbial communities were evaluated in the four studied reactors by means of 16S rRNA gene-based T-RFLP and high-throughput amplicon sequencing (Figs. 4, 5). It is well known that while the T-RFLP allows for the detection and semi-quantification of only the most abundant species in a sample [24–26], taxon-specific resolution of the HTS is much higher [27]. Therefore, knowing this limitation, we were not surprised that the calculated species richness based on the T-RFLP results was relatively lower compared to the amplicon sequencing (Additional file 1: Table S1). Also the total terminal restriction fragments (T-RF) diversity was only around 50 % of that resulting from the HTS. Similar discrepancies between the two molecular approaches were previously observed for AD reactors [27] as well as other studied environments, e.g. human anterior nares [28]. Additionally these differences can result from the use of different primers pairs for T-RFLP and HTS [29]. Due to the length limitation of the HTS technology, longer amplicons required for T-RFLP were too large for HTS. However, the calculated average pairwise Bray–Curtis distances reflecting the dissimilarities in community structures between the four reactors at the different sampling points were not significantly different between T-RFLP and HTS results (Fig. 6c, f). This observation suggests that both techniques accurately characterized the species dynamics over time in the four reactors, even though HTS was superior to T-RFLP regarding its sensitivity to detect minority species and the direct taxonomic affiliation of resulting sequencing reads. Interestingly, based on the HTS results, the ten most abundant bacterial operational taxonomic units (OTUs) at the sampling period P0 accounted for an average of 52.0 % ± 7.9 of the population in the studied reactors. At the same time the average bacterial richness calculated based on the T-RFLP was 8.7 ± 1.3, suggesting that indeed the T-RFLP approach could be sufficient to characterise at least the dominant part of the bacterial community in the reactor. Nevertheless, the attempts to in silico taxonomically assign the obtained T-RFs failed to provide unequivocal results due to a difference of a few base pairs. This discrepancy is often found between T-RFs predicted in silico and T-RFs measured in vivo [27, 30]. It usually results from the presence of unknown species in analysed environmental samples, thus the adequate 16S rRNA gene sequences lack in required databases used to assign in silico the generated T-RFLP results.

### Microbial communities in replicate reactors in steady state

At P0, on average, 22.8 % ± 5.7 and 63.3 % ± 13.8 of bacterial and below 1.0 % ± 0.0 and 19.6 % ± 3.3 of archaeal 16S rRNA amplicon reads could not be classified at the family and genus levels, respectively, confirming that largely bacterial and to a lesser extent archaeal communities in AD reactors remain uncharacterized. Both at the OTUs and T-RFs levels, bacterial and archaeal communities from replicate reactors (except for R2) clustered together at P0 based on non-metric multidimensional scaling (NMDS) analyses (Fig. 6). This indicates that stable operational conditions during the acclimation period led to similar microbial population in replicate AD reactors. In general, archaeal communities were much less diverse than bacterial ones (Additional file 1: Table S1), with *Methanomicrobiaceae*, *Methanobacteriaceae* and *Methanosaetaceae* being the dominant families at P0. Bacterial communities were dominated by representatives of the phyla *Bacteroidetes*, *Firmicutes* and candidate phylum *WWE1* (Fig. 5) with, respectively, *Porphyromonadaceae*, *Lachnospiraceae* and *Cloacamonaceaea* being the dominant families. The prevalence of *Bacteroidetes* and *Firmicutes* is not surprising and has been frequently reported for different AD reactors treating agricultural and agro-food residues (e.g. [31]). The other phyla including *Chloroflexi* and *Synergistetes* were only detected with low abundance. Concerning R2, the unexpected failure of the heating system during the acclimation period (exactly 239 days before P0; Fig. 1) leading to a reactor temperature drop to 30 °C for less than 24 h most probably resulted in a more dissimilar microflora, with *Thermotogae* and *Bacteroidetes* being the dominant bacterial phyla at P0. The archaeal community in R2 at P0 was dominated by *Methanoculleus* sp. (over 50 % of all 16S rRNA reads) and to a lesser extent by *Methanosaetasp.* and unknown *Methanospirillaceae* (Fig. 5).

Towards the end of the acclimation period, the HRT was gradually decreased in the four CSTRs, from more than 300 days at P0 to 28.5 days at P1 (data not shown), what influenced the shift of microbial diversity between the two sampling periods studied (Figs. 4, 5). While, at day 221 the OLR started to be gradually increased for the test reactors, it remained unchanged for CR, what resulted in a relatively stable bacterial community between P1 and P5 for this control reactor (Figs. 4, 5). The six prevalent bacterial T-RFs (T-RFs 33, 83, 91, 147, 166 and 373 bp), with high relative abundance at P1, were also detected for the other sampling points (Fig. 4). Similarly, the archaeal community remained stable in CR between the sampling periods P1 and P4, with the T-RF 107 bp representing more than 80 % of the total T-RF abundance (Fig. 4). At P5, its abundance decreased and another T-RF 106 bp started to emerge. By correlating 16S rRNA gene-based T-RFLP and 16S rRNA amplicon sequencing results, we could relate T-RF 107 bp to *Methanosaeta* sp., and T-RF 106 bp to *Methanoculleus* sp. The apparent redirection from acetoclastic towards hydrogenotrophic methanogenesis, as could be concluded based on the increased abundance of *Methanoculleus* sp. at P5, is most probably attributable to the depleting source of acetate (Fig. 2c), what is a direct consequence of the 11-day-long starving regime applied to CR (corresponds to the late acidosis and partially to the recovery period applied to R1–R3).

### Comparison of bacterial composition in replicate reactors exposed to increasing OLR and acidosis

As the strength of selection increased, due to the increased OLR applied to test reactors, the environment turned from a steady-state to a selective one and distinct microbes begun to dominate in the different R1–R3 reactors (Figs. 4, 5). Replicate test reactors developed thus their own bacterial populations that had their community structures more similar between the different sampling points for the same reactor, than between the different reactors for the same sampling point (Fig. 6a, b). At the same time, these communities were functionally redundant, since all reactors operated stably and showed similar global characteristics (Figs. 2, 3). Similarly to CR, the three dominant phyla, namely *Bacteroidetes,Firmicutes* and candidate phylum *WWE1* also dominated in R2–R3 (Fig. 5). No data for R1 could be recorded during the acidosis phase due to poor quality of extracted DNA for these sampling periods (harsh acidic environment). While in total, 441 OTUs were characterized for bacteria, roughly 50 top OTUs represented on average 88.1 % ± 4.4 of the whole bacterial populations in the studied reactors, and they were assigned to 12 different phyla (Fig. 5). Top 10 OTUs accounted for 70.8 % ± 14.9 of all sequenced 16S rRNA reads for the sampling periods P1–P5 for the four reactors, with the dominant representing the candidate phylum *WWE1* (OTU_1), *Bacteroidetes* (OTU_2, 3 and 5), *Firmicutes* (OTU_6, 7 and 18), the candidate phylum *OP9* (OTU_13) and *Synergistetes* (OTU_11). *Thermotogae* (OTU_4) was also dominant in case of R2.

In general, the diversity of *Firmicutes* with a total of 231 different OTUs (223 assigned to *Clostridia*) was much higher than *Bacteroidetes* with only 59 OTUs. While the dominant *Bacteroidetes* OTUs (they accounted for an average of 31.6 % ± 8.8 of the total bacterial 16S rRNA reads for R1–R3) appeared resistant to the increasing OLR and acidosis, *Firmicutes* (on average they accounted for 6.1 % ± 3.6 of total 16S rRNA reads for R1–R3) were much more sensitive (Additional file 2: Table S2). Several dominant *Firmicutes* OTUs disappeared (e.g. OTU_7, 15 and 32 unclassified bacteria of the order *MBA08*, OTU_14, an unclassified bacterium of the order *SHA*-*98*), and other completely new OTUs appeared during the acidosis. These new OTUs, e.g. OTU_18, 31, 78, 101 and 103, were classified as *Clostridiales*, including families of *Ruminococcaceae* and *Lachnospiraceae* (Additional file 2: Table S2). Whether this functional redundancy of *Firmicutes* explains their much higher diversity than *Bacteroidetes* is not clear at this stage and needs further investigation. Interestingly, it is well recognized that more diverse microbial communities provide a wider range of parallel pathways, what in principle should ensure their overall functional stability when confronted with an environmental stress [6]. That is why, diversity index is often proposed as a warning indicator of a process performance (see below). However, the fact that our reactors were dominated by a few resistant *Bacteroidetes* OTUs, suggests that each of these microbes must have a suite of parallel pathways encoded on its genome to allow to quickly adapt to the changing environment. Therefore, further metagenomic studies should show whether these yet uncharacterized *Bacteroidetes* are indeed broad-specificity bacteria.

The remaining phyla detected in our test reactors were much less diverse, with a candidate phylum *WWE1* being represented by five OTUs and *Thermotogae* by two OTUs only. The most abundant OTU_1, in terms of total 16S rRNA reads, was assigned to the candidate phylum *WWE1* and constituted a fourth part of the whole bacterial community in R2 and R3 between P1 and P4 (on average 27.6 % ± 3.9), and it only decreased during the recovery stage (13.6 % ± 1.5). For the test reactor R1 its abundance varied depending on the experimental stage. Interestingly, this OTU was below the detection limit for all reactors at the beginning of the experiment (P0). Candidatus *Cloacimonas* belonging to candidate division *WWE1* was previously shown to be the most dominant genus in an AD reactor co-digesting fish waste and cow manure [32]. The reconstructed genome of a representative bacterium from the same division suggested that it could be a hydrogen-producing syntroph [33]. Additionally, the involvement of the *WWE1* candidate bacteria to the fermentation of sugars was further confirmed by a high-resolution nanometer-scale secondary ion mass spectrometry (nano SIMS) [34]. With regards to *Thermotogae*, members of this phylum have been previously detected in mesophilic anaerobic reactors [35] and their presence could not be clearly linked to process parameters such as HRT or OLR [36]. Surprisingly, the high abundance of OTU_4 (*Thermotogae*) in R2 coincided with the higher pH reached by this reactor compared to the other test reactors during the late acidosis (P4, pH 5.7, Fig. 2g). Moreover, members of *Thermotogae* have been characterized for complex polysaccharide fermentation and hydrogen production [37], what might promote beneficial associations with hydrogenotrophic methanogens [38].

### Bacterial richness, diversity and evenness

Although the richness, evenness and diversity indices are often used to interpret the functioning of the reactor in terms of its microbial communities [39–42], their use as warning indicators remains questionable [6]. In principle, functionally diverse microbial communities provide a better suite of resistant, redundant and resilient pathways, and a higher microbial diversity is usually correlated with well-performing AD reactors [6]. However, when a stable (stochastic) environment faces a disturbance, it changes to a deterministic one, and the better adapted competitors begin to dominate, what is usually reflected by a decreased richness and diversity. Indeed, between the early and the late acidosis periods (P3 and P4), bacterial richness and diversity indices decreased for R1 and R3 (based on T-RFLP and HTS; Additional file 1: Table S1). In contrast, for the test reactor R2 these indices either increased (T-RFLP) or remained unchanged (HTS). Interestingly, while in general R2 was characterized by a lower species richness and diversity during the acidosis period than R3 (no HTS data for R1; Additional file 1: Table S1), it maintained the highest pH at P4 (5.7 vs 4.9 and 5.2 for R1 and R3), and produced significantly more biogas than R1 and R3, with the most favourable CH_4_/CO_2_ ratio (Figs. 2, 3).

While the use of richness and diversity parameters has still to be carefully considered, a decrease of evenness in structure of microbial diversity has been shown to be a good candidate warning indicator of process perturbation [43, 44]. Nevertheless, in our study the differences in the calculated evenness, based on the Pielou index (Additional file 1: Table S1), for R1–R3 and measured at the different sampling periods, did not show any uniform patterns, based on both T-RFLP and HTS results, suggesting that this index should also be carefully interpreted when used as a warning indicator. The overall functional redundancy of microbial communities demonstrated for our replicate overfed reactors, and the absence of uniform patterns in terms of the microbial ecology parameters raise the question whether the conclusions of the studies where only a single reactor was investigated (no replicates) are meaningful. Indeed, similarly to our observations, recent studies of parallel reactors treating beet silage [36] and co-digesting fish waste and cow manure [32], showed as well that distinct reactors can establish different microbial communities.

### Adaptation of archaeal population to acidosis and correlation with reactor’s parameters

**Fig. 7:**
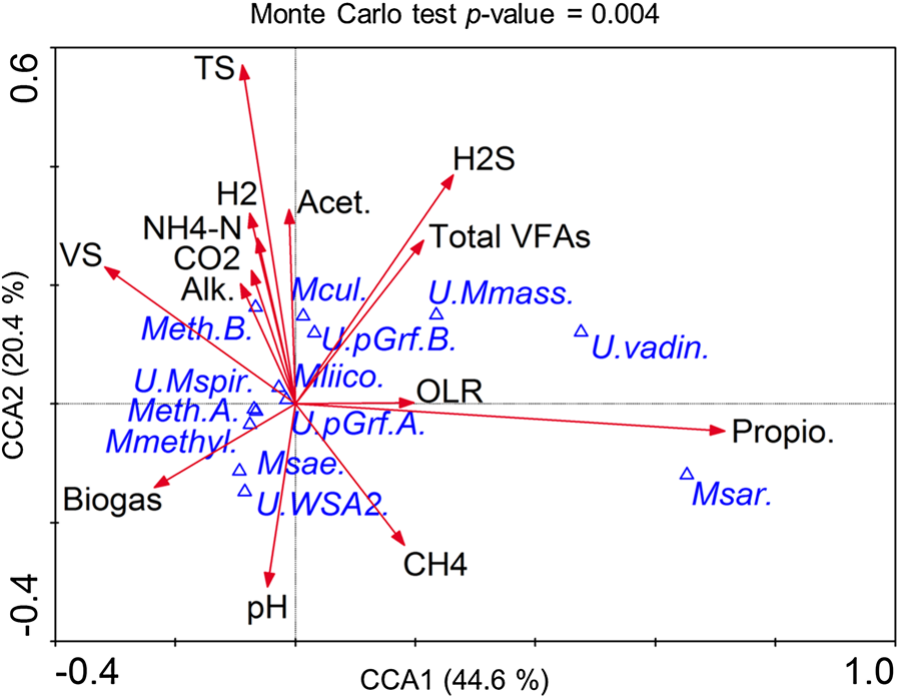
Canonical correspondence analysis (CCA) ordination diplot for the archaeal community. *Red vectors* represent the influence of the process parameters such as pH (pH), organic loading rate (OLR), biogas production (Biogas), total solids (TS), volatile solids (VS), methane (CH_4_), carbon dioxide (CO_2_) and hydrogen sulphide (H_2_S) contents in the biogas, alkalinity (Alk.), ammonium–nitrogen (NH_4_–N), total volatile fatty acids (Total VFAs), acetate (Acet.) and propionate (Propio.), contents in the sludge; *blue triangles* represent archaeal taxa derived from the high-throughput 16S rRNA amplicon sequencing at the species level: unknown *Methanomassiliicoccaceae* (*U.Mmass.*), unknown *Methanospirillaceae* (*U.Mspir*.), unknown *WSA2* (*U.WSA2.*), unknown *vadinCA11* (*U.vadin*.), *Methanobacterium* sp. A (*Meth.A*.), *Methanobacterium* sp. B (*Meth.B.*), *Methanoculleus* sp. (*Mcul*.), *Methanomassiliicoccus* sp. (*Mliico.*), *Methanomethylovorans* sp. (*Mmethyl.*), *Methanosaeta* sp. (*Msae.*), *Methanosarcina* sp. (*Msar.*), unknown *pGrfC26* A (*U.pGrf.A*), unknown *pGrfC26* sp. B (*U.pGrf.B*). A detailed correlation matrix including all process parameters and the archaeal community diversity is provided as Additional file 2: Table S4

Following the accumulation of VFAs and the resulting decrease of pH, and in contrast to another report [45], the richness and the diversity of the archaeal community were in general higher during the acidosis (P3 and P4) than at the beginning of the overfeeding campaign (P1) for the test reactors. The calculated evenness (Pielou index) for the archaeal population showed a trend similar to the bacterial community (Additional file 1: Table S1), providing no evidence that a lower evenness could be associated with a process imbalance. In continuation a canonical correspondence analysis (CCA) was used to highlight the influence of the changing process parameters on the archaeal community (CCA performed for bacteria was not statistically significant most probably due to the differential answer of the different communities that established in replicate reactors; *p* value of the Monte Carlo test was >0.05; therefore, these results are not discussed here). As shown in Fig. 7, 65 % of the total species variance could be explained by the first two axes of the CCA ordination diplot. While pH showed a strong positive correlation with the alkalinity (*r* = 0.94, *p* value <0.001) and a negative correlation with the VS content (*r* = −0.89, *p* value <0.001), it was uncorrelated with the total biogas production (*p* value = 0.34). This may be explained by the fact that total biogas production was not strongly reduced during acidosis, due to the increased production of CO_2_ and most probably H_2_ (its correct concentration could not properly be measured due sensor saturation; Fig. 3) by hydrolytic and acidogenic bacteria. This is well reflected by a strong negative correlation between pH and CO_2_ content in the biogas (*r* = −0.80, *p* value <0.001). At the same time, pH also showed a strong positive correlation with the methane content in the biogas (*r* = 0.80, *p* value <0.001), which was significantly reduced during the acidosis (Fig. 3d–f). Interestingly, higher biogas production was also correlated with the abundance of *Methanosaeta* sp. (*r* = 0.48, *p* value <0.05). Moreover, the presence of *Methanosaeta* sp. was negatively correlated with the total VFAs content in the reactors (*r* = −0.72, *p* value <0.001) and with the level of abundance of *Methanoculleus* sp. (*r* = −0.49, *p* value < 0.05). Indeed, in the overfed reactors R1–R3, the relative abundance of T-RFs 107 bp, assigned to *Methanosaeta* sp., was dominant at the beginning of the OLR increase (P1), then decreased toward acidosis and was replaced by T-RFs 106 pb, related to *Methanoculleus* sp. (Figs. 4, 5). This suggests an overall redundancy of the archaeal population in sub-optimal conditions. Similarly, a low pH and high VFAs-determined transition from an acetoclastic (*Methanosaeta* sp.-dominated) toward a hydrogenotrophic (*Methanoculleus* sp.-dominated) methanogenesis has been previously reported for an anaerobic membrane bioreactor treating swine manure and exposed to high shear condition [46], and for a hybrid anaerobic reactor exposed to the changing OLR [45]. Even though, in the absence of *Methanosaetaceae*, syntrophic acetate oxidation (SAO) and hydrogenotrophic methanogenesis were proposed to be the dominant methane-producing pathways from acetate [47], we did not detect any of a few known SAO bacteria in our test reactors [44] (Additional file 2: Table S2). This might indicate either the presence of previously unknown SAO bacteria, which could be expected regarding a relatively high number of taxonomically unassigned 16S rRNA sequences generated in this study, or the existence of new metabolic pathways leading to VFAs conversion and a subsequent methane production.

*Methanosaeta* sp. is regarded as a redundant archaeon and a decrease in its population, or more generally a disturbance of a stable archaeal population has been proposed as an early warning indicator of process failure [6]. Here, by correlating the 16S rRNA amplicon sequencing with the changes observed at macroscopic level, we could observe that indeed a decrease of *Methanosaeta* sp. population and its substitution by *Methanoculleus* sp. started in the test reactors at the sampling period P2. For the same, the pH was still above 7, no accumulation of VFAs was observed, and the methane yield was not yet affected (Figs. 2, 3).

Even though, the dominance of *Methanoculleus* was documented for many well-performing farm reactors [48], it is not the presence of this species (e.g. P0 in our studied reactors) which could point to the approaching process disturbance, but the sudden shift of the dominant population in steady-state reactors. Therefore, and in accordance to other authors [6], we tentatively propose that substitution of a dominant archaeal population (*Methanosaeta* sp. is often dominant in reactors inoculated with sludge from a wastewater treatment plant (WWTP) and operated at low acetate and free-ammonia concentrations [48–51], Fig. 7) with another population of archaea (*Methanoculleus* sp. emerged at the onset of VFAs accumulation) could indeed be regarded as an early warning indicator of acidosis. Nevertheless, other authors propose other e.g. *Methanosarcina thermophilia*-related species, characterized as heavy duty methanogens [49], to be the earliest indicators of acidification in overloaded fed-batch biogas reactor digesting maize silage [52]. In our study *Methanosarcina* sp. was not detected neither before nor during the acidosis (Fig. 5). Therefore, we suggest to conduct further large-scale monitoring of different well-performing as well as failed AD reactors fed with diverse substrates before a microbial species can unequivocally be proposed as a good and universal indicators of any process imbalance.

### Restoration of biogas production with NaOH and NaHCO_3_ addition

A starving period and a gradual addition of NaOH and NaHCO_3_ to R1–R3 following the acidosis, resulted in a pH increase to a neutral level after 3 days only. The complete process recovery and restoration of biogas production with the methane content exceeding 50 %, occurred already after 9 days following the beginning of the alkali addition. In comparison, bioaugmentation of overfed acidic reactors (pH decreased from around 7.0 to 6.8 only), has been shown to decrease the recovery time by a maximum of 37 days, compared to about 180 days for non-bioaugmented ones [53]!

Interestingly, the archaeal population in the test R1–R3 reactors, although affected in terms of community structure dynamics, was not eradicated by a relatively low pH (5.2 ± 0.4) during the acidosis. Following the pH increase, and probably as a consequence of starvation and NaOH influence on archaeal population, *Methanosarcina* sp. which was also described as a fast growing archaeon resistant to high acetate concentration [49], started to outcompete *Methanoculleus* sp. in R2 and R3 (Fig. 5). Remarkably, a new archaeal OTU_15 classified as a candidate genus *vadinCA11* that was below the detection limit in previous periods, emerged in R1 and R2 during the recovery phase (P5) (Fig. 5; Additional file 2: Table S3). This genus has been reported as potentially halophilic [54] and it is probable that the increase of sodium ion concentration due to the addition of the alkali favoured its outbreak during the recovery phase.

A drastic pH increase influenced the structure of the dominant bacterial phyla as well. While the three dominant *Bacteroidetes* OTUs (OTU_2, 3 and 5) at P1–P4 were still detected at P5, however, with lower abundance, new OTUs (OTU_19, 45, 65 and 74, Additional file 2: Table S2) also representing families of *Porphyromonadaceae* and *Bacteroidaceae* started to be detected at high level inside the *Bacteroidetes* population during the recovery phase P5. *Firmicutes* also answered with a functional redundancy to the pH change. Three OTUs (OTU_25, 68 and 72, Additional file 2: Table S2) emerged and became dominant (OTU_25 assigned as unclassified *Sedimentibacter*, OTU_68 potentially assigned as *Clostridium bolteae* and OTU_72 assigned as unclassified *Epulopiscium*). At P5, OTU_68 constituted 10.6, 0.5 and 7.6 % of the whole bacterial population, based on the HTS results, respectively, for R1, R2 and R3. *C. bolteae*, which is a spore-forming propionate producer, was only detected for R3 at P0 and at a very low abundance level (around 0.01 % of the whole bacterial community). As one could expect, an increased propionate concentration at P5 was linked to an increased abundance of a propionate-producing *C. bolteae* and a decrease in abundance of potentially propionate-consuming *WWE1* candidate bacteria [33]. Although the bacterial and archaeal community structures completely changed for the three reactors R1–R3, following the alkali addition (Figs. 4, 5), the microbial population successfully self-optimized to the new environmental conditions, as the biogas production restarted quickly (Fig. 3a). On the one hand we showed that the addition of NaOH to raise the pH and NaHCO_3_ to raise the alkalinity quickly restored the activity of overfed reactors; therefore, it represents an efficient treatment of AD reactors that faced acidosis. On the other hand, the drastically changing environmental conditions influenced the structure of the prevailing microbial communities in a way that a favourable niche was created for other species to emerge.

## Conclusions

The impact of increasing OLR of sugar beet pulp, acidosis and finally process recovery on microbial community richness and diversity in three mesophilic CSTR reactors (R1–R3) were compared with a cautiously fed reactor (CR). As major outputs of this study we could point out that even though the overfed reactors responded rather similarly in terms of process performance to the OLR increase, each reactor established its own microbial community structure over time, dominated only by a few OTUs. The retrospective management [6] of our reactors showed that traditional microbial parameters such as richness, diversity and evenness which could be potentially used to predict process imbalances were not effective, due to the differential behaviours of microbial consortia in the replicated reactors. Moreover, the reactor that was characterized with the least diverse microbial community (R2) was the most resistant to acidosis and the best performing one in terms of methane production. The shift from *Methanosaeta* sp. to *Methanoculleus* sp. in *Methanosaeta* sp.-dominated reactor could be an interesting and promising warning indicator of an approaching acidosis since this shift occurred before pH started to decreased. The combination of the reactor starvation and the pH adjustment with NaOH and NaHCO_3_ demonstrated a good potential for process recovery after acidosis, as reflected by a quick restart of the methane production, even if a new microbial community has established. Nevertheless, longer monitoring periods of microbial communities after man-induced process recovery are necessary to evaluate if the newly established microbial populations are stable over time and if they are better adapted to high VFAs concentrations. Relying on the results of this study, alkali-mediated AD process recovery from acidosis was successfully applied to two full-scale anaerobic reactors in Germany and Luxembourg (data not shown).

## Methods

### Reactors operation, monitoring and sampling

Four anaerobic pilot-scale CSTR reactors were inoculated with seeding sludge originating from a mesophilic AD reactor of a WWTP located in Schifflange, Luxembourg (SIVEC-Schifflange). To be the closest as possible to a full-scale anaerobic reactor conditions, the working volume of our lab CSTR was 100 L. Each reactor was equipped with an individual temperature regulation system to assure a constant temperature of 37 ± 0.2 °C and pH was measured daily with a pH 196 Microprocessor pH meter connected to a SenTix^®^ 21 pH electrode (WTW, Weilheim, Germany). The sludge total solids (24 h at 105 °C) and volatile solids (6 h at 550 °C) were determined on a weekly basis, according to the 4630 VDI norm [55]. Alkalinity (mg CaCO_3_ L^−1^ of sludge) and ammonium–nitrogen concentration (kg NH_4_–N m^−3^ of sludge) were measured weekly, in conformity with the manufacturers’ protocol, using the BiogasPro system (RIMU, Königsbrunn, Germany). The concentration of VFAs was measured following an ether extraction and using a gas chromatograph (Agilent technologies, Santa Clara, USA) equipped with a Varian CP-FFAP column and a flame ionization detector (FID). The migration was done with helium (He) as a carrier gas. The total VFAs concentrations (mg kg^−1^) were expressed as the sum of the individual volatile fatty acids concentrations, measured for acetate, propionate, isobutyrate, butyrate, isovalerate, valerate and caproate. Four individual gas counters of the wet-drum type of 1 mL resolution (Ritter, Bochum, Germany) were used to continuously monitor the gas production for each reactor. The gas production measurement was cumulated to produce hourly data and expressed in litres, at normal atmospheric pressure and temperature conditions (NL). Gas quality (CH_4_ and CO_2_ content) was measured online every 2 h with dedicated nondispersive infrared sensors in the 0–100 % volume measurement range (Dynament, South Normanton, UK). The concentration of H_2_S and H_2_ was measured with an electrochemical sensor (in the 0–10,000 ppm range, ITG, Wismar, Germany) and a specific 65-2440RK sensor (0–2000 ppm range; RKI Instruments Inc., Union City, USA), respectively, on the same time basis as CH_4_ and CO_2_.

Following the reactors inoculation and during a 16-month-long adaptation phase (data not shown) the four reactors were fed every working day with a mono-substrate, using sugar beet pulp pellets [86.8 % total solids (TS), 92.0 % volatile solids (VS), C/N: 39.63] at the OLR of 1.60 kg VS m^−3^ day^−1^, to increase the TS content of the original sludge from about 2 % to 7–8 % (TS content commonly observed in farm biogas reactors).To best mimic traditional full-scale anaerobic reactors, the added substrate was not sterilized. In continuation, the feeding was adapted to maintain a constant HRT of 28.5 days by mixing the sugar beet pulp with tap water. Once a stable TS content was obtained, the sludge was sampled (sampling period zero: P0, day 1) and the experimental campaign was initiated (Fig. 1). Three reactors (overfed reactors: R1, R2, R3) were further fed with an increasing OLR, while one reactor was used as a control (cautiously fed reactor; CR), and was constantly fed at an OLR of 1.60 kg VS m^−3^ day^−1^ (Fig. 1a). The choice of the experimental design present in this study (three replicated overfed reactors versus only one control reactor) was dictated by the fact than only four reactors of 100 L capacity were available; therefore, we decided to give priority for data generation to the overfed reactors. The OLR for R1-R3 was gradually increased by increments of 0.40 kg VS m^−3^ every week until reaching acidosis (Fig. 1b). To promote the process recovery, the feeding was stopped at day 267. After 6 days of starving under very acidic pH (5.2 ± 0.4), the pH was artificially increased to reach a value of 7 by adding 7, 2 and 5 L of 1 M NaOH to R1, R2 and R3, respectively (each reactor requiring specific amount of alkali). In continuation, NaHCO_3_ dry salt was slowly added to increase the alkalinity (around 900, 600 and 800 g for R1, R2 and R3, respectively) until a pH of 7.5 was reached. The three test reactors were then starved for 5 more days. The total starving of 11 days was also applied to the CR. Finally, after the 11 days of starving, as the methane content in the biogas reached again at least 50 % for R1–R3 (Fig. 3), the feeding was re-initiated for all the reactors at an OLR of 1.60 kg m^−3^ day^−1^. Sludge aliquots for R1–R3 and CR were sampled for six periods P0–P5 (Fig. 1) and stored frozen at −20 °C prior the microbial community analyses.

### Microbial monitoring by 16S rRNA gene-based T-RFLP analysis

Genomic DNA was extracted as previously described by Klocke et al. [56]. Bacteria-specific 16S rRNA gene forward primer 27f, labelled with 6-carboxyfluorescein (6-FAM), and the reverse primer 926MRr (Table 1; Eurogentec, Seraing, Belgium) were used for amplification with the following PCR conditions; initial denaturation at 98 °C for 3 min, followed by 25 cycles of denaturation at 98 °C for 20 s, annealing at 54 °C for 15 s, elongation at 72 °C for 1 min and a final elongation at 72 °C for 7 min. To amplify the archaeal 16S rRNA gene, forward primer Ar109f labelled with 6-FAM and the reverse primer Ar912r (Table 1; Eurogentec, Seraing, Belgium) were used with the following PCR conditions: initial denaturation at 98 °C for 3 min, followed by 29 cycles of denaturation at 98 °C for 20 s, annealing at 55 °C for 15 s, elongation at 72 °C for 1 min, and a final elongation at 72 °C for 7 min. Amplifications were done using a TProfessional Thermocycler (Biometra GmbH, Goettingen, Germany) and the Phusion^®^ Taq polymerase (New England Biolabs Inc., Ipswich, USA) in a final volume of 25 µL with 0.4 µM of each primer and 0.5 µL of a 1:10 and 1:50 dilution of the DNA extracts, respectively, for bacteria and archaea. PCR products were purified with the QIAquick PCR Purification Kit (Qiagen, Hilden, Germany) according to the manufacturer’s protocol. In continuation, 100–200 ng of purified bacterial 16S rRNA amplicons were digested at 37 °C for 4 h with the *Msp*I and *Hin*6I restriction enzymes (Thermo Fisher Scientific, Waltham, USA). For Archaea, between 200 and 500 ng of PCR products were digested at 37 °C for 4 h with the *Alu*I restriction enzyme (Thermo Fisher Scientific, Waltham, USA). Digested products were precipitated with 0.1 v/v of sodium acetate (3 M) and 10 v/v of ethanol (75 %). Samples were incubated for 30 min in the dark and at room temperature, and centrifuged at 14,000 rpm at 4 °C for 30 min. Following the centrifugation, the supernatant was discarded, 10 v/v of ethanol (75 %) was added and samples were incubated for a second time during 10 min in the dark and at room temperature; and centrifuged at 14,000 rpm and 4 °C for 30 min. Finally, following the centrifugation, the supernatant was discarded, samples were dried at 37 °C, re-suspended in 40 µL of sterile ultrapure water and incubated at 55 °C for 15 min at 300 rpm. In continuation, samples were mixed with 10 µL of Hi-Di formamide (Applied Biosystems, Stafford, USA) containing 1:250 (v/v) carboxy-X-rhodamine (ROX)-labelled MegaBace™ ET900-R size standard (GE Healthcare, Piscataway, USA). Ultrapure water was added to reach the final volume of 16 µL. Samples were denatured at 95 °C for 3 min and directly placed on ice. The sizes of generated DNA fragments were determined using an Applied Biosystems 3130 Genetic Analyser (Applied Biosystems, Stafford, USA) (POP 7 matrix, capillary size: 50 cm) with the following parameters: injection voltage, 3.0 kV; injection duration, 12 s; run voltage, 8.5 kV; run time at 60 °C, 5500 s. The GeneMapper software (version 4.0; Applied Biosystems, Stafford, USA) was used to analyse the T-RFLP chromatograms. Fragments with fluorescence intensity below 200 relative fluorescence units were discarded from further analysis and following Dunbar et al. [57], the total fluorescence of each sample was standardized three times to the smallest quantity. Online T-Rex software was used to align the T-RFs with a clustering threshold of 0.8 [58]. The length of each T-RF was expressed in base pair (bp). Figure 4 shows the results of the 16S rRNA gene-based T-RFLP analysis, with the graphs representing an average of three technical replicates performed for each sample.

**Table 1:** PCR primers targeting the 16S rRNA genes of the bacterial and archaeal community

Primer	Direction	Sequence [5′ → 3′]	Targeted domain	References
27f	Forward	AGA GTT TGA TCM TGG CTC AG	Bacteria	[66]
926MRr	Reverse	CCG TCA ATT CMT TTR AGT TT	Bacteria	[67]
S-D-Bact-0341-a-S-17	Forward	CCT ACG GGA GGC AGC AG	Bacteria	[68]
S-D-Bact-0787-b-A-20	Reverse	GGA CTA CHV GGG TAT CTA AT	Bacteria	[68]
Ar109f	Forward	ACK GCT CAG TAA CAC GT	Archaea	[69]
Ar 912r	Reverse	CTC CCC CGC CAA TTC CTT TA	Archaea	[70]
S-D-Arch-0519-a-S-15	Forward	CAG CMG CCG CGG TAA	Archaea	[68]
S-D-Arch-1041-a-A-18	Reverse	GGC CAT GCA CCW CCT CTC	Archaea	[68]

### Microbial monitoring by high-throughput 16S rRNA amplicon sequencing

Genomic DNA extractions from samples of the six sampling periods P0–P5 were performed this time using the PowerSoil DNA Isolation Kit (Mobio Laboratories Inc., Carlsbad, CA, USA) in accordance with the manufacturers’ protocol. The 16S rRNA gene libraries were constructed with a modified primer pair S-D-Bact-0341-a-S-17 and S-D-Bact-0787-b-A-20 targeting a fragment of 466 bp of the bacterial V3–4 region and primer pair S-D-Arch-0519-a-S-15 and S-D-Arch-1041-a-A-18 targeting a fragment of 526 bp of the archaeal V4–6 region (Table 1). The modification included the incorporation in the 5′ end of the Nextera XT^®^ transposase sequence (Illumina Inc., San Diego, USA) in the forward and the reverse primer, and additional four N (i.e. four random nucleotides) in the forward primer to increase the nucleotide diversity. Amplicons were generated using the Q5^®^ Hot Start High-Fidelity DNA Polymerase (New England Biolabs Inc., Ipswich, USA), purified with the AMpure magnetic beads (Agencourt, Beckman Coulter Inc., Fullerton, USA) and quantified with the Qubit^®^ dsDNA HS assay kit (Life technologies, Carlsbad, USA). The concentration of the amplicons was adjusted to 1 ng µL^−1^ and 1 µL of each library was used as a template in a second PCR where the Nextera XT^®^ barcodes and the Illumina adapters necessary for hybridization to the flow cell were added with the Nextera XT Index kit. The resulting amplicons were purified with the AMpure magnetic beads (Agencourt) and pooled in equimolar concentrations. The final concentration of the library pool was determined with a KAPA SYBR^®^ FAST Universal qPCR Kit (Kapa Biosystems, Wilmington, USA). Libraries were mixed with Illumina-generated PhiX control libraries (5 %) and sequenced with the MiSeq Reagent Kit V3-600 cycles. The obtained sequence reads were de-multiplexed, quality trimmed and assigned to OTUs at 97 % similarity with Usearch (v7.0.1090_win64) pipeline [59]. Taxonomy affiliation was done with the Greengenes database (http://greengenes.lbl.gov/) and the final nucleotide sequences obtained were deposited in the GenBank database (http://www.ncbi.nlm.nih.gov/genbank/) under accession numbers KR013301 to KR013741 for bacteria and KR013288 to KR013300 for archaea.

### Data analysis and statistics

Richness (numbers of distinct T-RFs and OTUs) and diversity indices, including Shannon–Weaver index (*H*′) [60] which measures the entropy, Pielou index (*J*) [61] which measures the evenness, i.e. the equitability between the different species present in a community, were calculated based on the microbial community analyses results. To assess the richness and diversity indices over time, linear mixed-effect models (LMM) were calibrated for each reactor, considering time (the different sampling points) as fixed factor and the replicates as random factor. Post hoc tests (Tukey’s honest significant difference test) were applied to further compare pairwise sampling points for significant LMM. To compare, respectively, the bacterial and archaeal community results from the two molecular techniques (T-RFLP and HTS) used in this study, pairwise Bray–Curtis [62] distance matrices were calculated using Mothur [63] and represented using NMDS. The average pairwise Bray–Curtis distance for each microbial community between results from T-RFLP and HTS were then compared statistically using Wilcoxon signed-rank test. Differences were considered statistically significant at a *p* value <0.05. The influence of process parameters on the microbial community diversity, assessed by HTS, was analysed using CCA with the CANOCO software (version 4.5) [64] and a correlation analysis (Spearman). The significance test for CCAs was carried out by Monte Carlo permutation (499 times) and correlations were considered significant at a *p* value <0.05. All statistical and correlation analyses were performed using the freeware R version 3.1.0 [65].

## Additional files

Additional file 1:
**Table S1.** Community diversity and evenness indices for the four reactors CR, R1–R3. Additional file 2:
**Table S2.** Abundance levels and taxonomic affiliation of bacterial OTUs. **Table S3.** Abundance levels and taxonomic affiliation of archaeal OTUs. **Table S4.** Spearman correlation matrix for the relationships between the process parameters and the archaeal community diversity.

## Abbreviations

AD: anaerobic digestion
CCA: canonical correspondence analysis
CHP: combined heat and power unit
CR: control (cautiously fed) reactor
CSTR: continuously/completely stirred tank reactor
*H*′: Shannon–Weaver index
HRT: hydraulic retention time
HTS: high-throughput amplicon sequencing
*J*: Pielou index
LMM: linear mixed-effect models
nano SIMS: nanometer-scale secondary ion mass spectrometry
NMDS: non-metric multidimensional scaling
OLR: organic loading rate
R1–R3: test (overfed) reactors R1, R2 and R3
SAO: syntrophic acetate oxidation
T-RF: terminal restriction fragment
T-RFLP: terminal restriction fragment length polymorphism
TS: total solids
VFAs: volatile fatty acids
VS: volatile solids
OTU: operational taxonomic unit
WWTP: wastewater treatment plant
