# Supplementary material for: Microbial community dynamics in replicate anaerobic digesters exposed sequentially to increasing organic loading rate, acidosis, and process recovery
Source: Biotechnol Biofuels. 2015 Aug 19;8:122. doi: 10.1186/s13068-015-0309-9 (PMC4539856; doi:10.1186/s13068-015-0309-9)
Supplement: Additional file 1: — Table S1. Community diversity and evenness indices for the four reactors CR, R1–R3. [file 13068_2015_309_MOESM1_ESM.docx]

# Additional file 1

### Table S1 - Community diversity and evenness indices for the four reactors CR, R1-R3

|  |  | CR | |  | |  | R1 | | | | | R2 | | | |  | R3 | | | |
| --- | --- | --- | --- | --- | --- | --- | --- | --- | --- | --- | --- | --- | --- | --- | --- | --- | --- | --- | --- | --- |
|  | Sampling period | | *H’* | *J* | *Richness* |  | | *H’* | *J* | *Richness* |  | | *H’* | *J* | *Richness* |  | | *H’* | *J* | *Richness* |
|  | P0 | 2.71 ± 0.20 a | | 0.87 ± 0.04 c | 8.7 ± 0.6 a |  | 2.96 ± 0.13 b | | 0.89 ± 0.00 d | 10.0 ± 1.0 ab |  | 2.37 ± 0.10 ab | | 0.85 ± 0.03 b | 7.0 ± 1.0 a |  | 2.63 ± 0.07 c | | 0.83 ± 0.02 b | 9.0 ± 0.0 b |
|  | P1 | 2.74 ± 0.05 a | | 0.81 ± 0.00 ab | 10.3 ± 0.6 a |  | 3.04 ± 0.03 b | | 0.88 ± 0.01 c | 11.0 ± 0.0 b |  | 2.27 ± 0.06 a | | 0.74 ± 0.01 a | 8.3 ± 0.6 b |  | 2.62 ± 0.03 c | | 0.83 ± 0.01 b | 9.0 ± 0.0 b |
| Bacteria | P2 | 2.62 ± 0.08 a | | 0.81 ± 0.00 ab | 9.3 ± 0.6 a |  | 2.69 ± 0.01 a | | 0.85 ± 0.00 b | 9.0 ± 0.0 a |  | 2.83 ± 0.02 c | | 0.82 ± 0.01 b | 11.0 ± 0.0 c |  | 2.48 ± 0.07 bc | | 0.86 ± 0.01 cd | 7.3 ± 0.6 a |
| T-RFLP | P3 | 2.73 ± 0.05 a | | 0.85 ± 0.01 bc | 9.3 ± 0.6 a |  | 3.01 ± 0.01 b | | 0.91 ± 0.00 e | 10.0 ± 0.0 ab |  | 2.47 ± 0.25 ab | | 0.83 ± 0.03 b | 8.0 ± 1.0 ab |  | 3.08 ± 0.00 d | | 0.89 ± 0.00 d | 11.0 ± 0.0 c |
|  | P4 | 2.45 ± 0.42 a | | 0.81 ± 0.03 ab | 8.3 ± 2.3 a |  | 2.65 ± 0.05 a | | 0.81 ± 0.01 a | 9.7 ± 0.6 a |  | 2.78 ± 0.08 c | | 0.82 ± 0.01 b | 10.3 ± 0.6 c |  | 2.16 ± 0.26 a | | 0.76 ± 0.02 a | 7.3 ± 1.5 a |
|  | P5 | 2.32 ± 0.01 a | | 0.77 ± 0.00 a | 8.0 ± 0.0 a |  | 3.21 ± 0.00 c | | 0.93 ± 0.00 f | 11.0 ± 0.0 b |  | 2.50 ± 0.05 b | | 0.85 ± 0.02 b | 7.7 ± 0.6 ab |  | 2.37 ± 0.02 ab | | 0.84 ± 0.00 bc | 7.0 ± 0.0 a |
|  | P0 | 4.74 | | 0.60 | 242 |  | 4.97 | | 0.62 | 263 |  | 3.54 | | 0.46 | 216 |  | 4.52 | | 0.57 | 247 |
|  | P1 | 4.09 | | 0.52 | 246 |  | 4.96 | | 0.57 | 239 |  | 4.08 | | 0.51 | 268 |  | 4.35 | | 0.55 | 248 |
| Bacteria | P2 | 4.22 | | 0.53 | 245 |  | 4.06 | | 0.53 | 203 |  | 3.54 | | 0.47 | 193 |  | 3.90 | | 0.50 | 216 |
| HTS | P3 | 4.04 | | 0.51 | 239 |  | - | | - | - |  | 4.04 | | 0.53 | 194 |  | 4.37 | | 0.57 | 212 |
|  | P4 | 3.42 | | 0.44 | 209 |  | - | | - | - |  | 3.90 | | 0.51 | 194 |  | 4.19 | | 0.55 | 205 |
|  | P5 | 4.06 | | 0.51 | 239 |  | 5.16 | | 0.65 | 249 |  | 4.24 | | 0.55 | 199 |  | 4.97 | | 0.63 | 229 |
|  | P0 | 1.09 ± 0.15 ac | | 0.52 ± 0.03 bc | 4.3 ± 0.6 a |  | 1.35 ± 0.00 ab | | 0.00 ± 0.00 a | 6.0 ± 0.0 ab |  | 0.58 ± 0.00 a | | 0.36 ± 0.00 a | 3.0 ± 0.0 ab |  | 1.21 ± 0.11 b | | 0.53 ± 0.05 bc | 5.0 ± 1.4 ab |
|  | P1 | 0.83 ± 0.07 a | | 0.41 ± 0.03 a | 4.0 ± 0.0 a |  | 1.17 ± 0.15 a | | 0.51 ± 0.00 b | 5.0 ± 1.0 a |  | 1.00 ± 0.07 a | | 0.50 ± 0.03 a | 4.0 ± 0.0 b |  | 0.84 ± 0.14 ab | | 0.42 ± 0.07 ab | 4.0 ± 0.0 a |
| Archaea | P2 | 1.10 ± 0.18 ac | | 0.49 ± 0.04 ab | 4.7 ± 0.6 a |  | 1.87 ± 0.29 bc | | 0.68 ± 0.08 c | 6.7 ± 0.6 ac |  | 1.80 ± 0.08 b | | 0.66 ± 0.04 b | 6.7 ± 0.6 bc |  | 0.61 ± 0.19 a | | 0.32 ± 0.07 a | 3.7 ± 0.6 a |
| T-RFLP | P3 | 1.31 ± 0.33 bc | | 0.53 ± 0.05 bc | 5.7 ± 1.3 a |  | 2.30 ± 0.33 cd | | 0.71 ± 0.05 c | 9.3 ± 1.5 b |  | 2.60 ± 0.64 c | | 0.80 ± 0.07 c | 9.7 ± 3.2 c |  | 1.72 ± 0.07 c | | 0.61 ± 0.02 cd | 7.0 ± 0.0 cd |
|  | P4 | 0.96 ± 0.11 ab | | 0.46 ± 0.03 ab | 4.3 ± 0.6 a |  | 1.80 ± 0.24 bc | | 0.61 ± 0.06 bc | 7.7 ± 1.5 bc |  | 2.31 ± 0.16 bc | | 0.79 ± 0.01 c | 7.7 ± 1.2 ac |  | 1.71 ± 0.22 c | | 0.59 ± 0.06 cd | 7.3 ± 0.6 d |
|  | P5 | 1.38 ± 0.21c | | 0.59 ± 0.09 c | 5.0 ± 0.0 a |  | 2.85 ± 0.08 d | | 0.87 ± 0.00 d | 9.7 ± 0.6 b |  | 1.94 ± 0.20 bc | | 0.68 ± 0.04 b | 7.3 ± 0.6 bc |  | 1.66 ± 0.15 c | | 0.67 ± 0.03 d | 5.7 ± 0.6 bc |
|  | P0 | 2.66 | | 0.80 | 10 |  | 2.77 | | 0.77 | 12 |  | 2.25 | | 0.65 | 11 |  | 2.66 | | 0.77 | 11 |
|  | P1 | 2.38 | | 0.72 | 10 |  | 2.48 | | 0.69 | 12 |  | 2.66 | | 0.84 | 9 |  | 2.30 | | 0.69 | 10 |
| Archaea | P2 | 2.22 | | 0.67 | 11 |  | 2.35 | | 0.66 | 12 |  | 2.53 | | 0.76 | 10 |  | 2.14 | | 0.60 | 12 |
| HTS | P3 | 2.28 | | 0.66 | 11 |  | - | | - | - |  | 2.69 | | 0.75 | 12 |  | 1.64 | | 0.46 | 12 |
|  | P4 | 2.37 | | 0.68 | 11 |  | - | | - | - |  | 2.67 | | 0.80 | 10 |  | 1.49 | | 0.40 | 13 |
|  | P5 | 2.11 | | 0.59 | 12 |  | 2.67 | | 0.84 | 9 |  | 2.24 | | 0.65 | 11 |  | 1.29 | | 0.35 | 13 |

*H’*: Shannon-Weaver index; *J*: Pielou index; P0-P5 are the sampling periods chosen for microbial community analysis; CR is the cautiously fed reactor; R1-R3 are the reactors exposed to increasing organic loading rate, T-TRFLP: results from the 16S rRNA gene-based T-RFLP analysis, HTS: results from the high-throughput 16S rRNA amplicon sequencing. Statistical analyses were performed using linear mixed-effects models combined to a pairwise test (Tukey) for results of the 16S rRNA gene-based T-RFLP analysis, figures holding the same letter in a column and for one taxonomic domain (Bacteria or Archaea) do not differ significantly (p ≤ 0.05); there were no replicate for the HTS analysis
